# Chiropractor Perceptions of Non-registered Practitioners Utilizing Spinal Manipulative Therapy in Hong Kong: A Cross-Sectional Survey

**DOI:** 10.7759/cureus.70734

**Published:** 2024-10-02

**Authors:** Wai Ting Lee, Eric Chun-Pu Chu, Wing Ka Cheng

**Affiliations:** 1 Chiropractic and Physiotherapy Centre, New York Medical Group, Hong Kong, CHN

**Keywords:** chiropractic, chiropractic therapy, hong kong chiropractor, public health, public health and safety

## Abstract

Objective

This study aimed to explore registered chiropractors' perceptions of the use of spinal manipulative therapy (SMT) by non-registered practitioners in Hong Kong, assessing the associated risks, adverse events, and patient management approaches.

Method

A cross-sectional survey was conducted among registered chiropractors in Hong Kong using an online questionnaire. The survey, distributed via chiropractic associations from January 26 to March 20, 2024, gathered data on the practitioners' experiences with patients previously treated by non-registered practitioners, including the types of adverse events observed and subsequent interventions required. Descriptive statistics were used to analyze the data. A total of 134 valid responses were received.

Results

Among respondents, a significant proportion (91%) of chiropractors reported observing adverse effects in patients treated by non-registered practitioners, with the most common issues being the aggravation of existing symptoms (95.5%) and new or unexpected complaints (82.8%). The most frequently required interventions included imaging (97%), medication (58.2%), and specialist referrals (59%). Respondents indicated that these adverse events could often have been prevented if proper training and certification were in place. Common reasons cited by patients for seeking care from non-registered practitioners included a lack of awareness about licensed care (90.3%) and cost considerations (80.6%).

Conclusion

The study highlights concerns reported by registered chiropractors regarding the safety of SMT performed by non-registered practitioners in Hong Kong. These concerns emphasize the need for enhanced public education on potential risks and stricter regulatory oversight. Although direct data on the occurrence of adverse events was not collected, the findings suggest that many risks could potentially be mitigated with proper training, underscoring the importance of credentialed care in ensuring patient safety.

## Introduction

The landscape of healthcare is continually evolving, with complementary and alternative medicine playing an increasingly integral role in patient care [1]. In the vibrant metropolis of Hong Kong, where traditional and modern medical practices coexist, the utilization of spinal manipulation by non-registered practitioners has emerged as a distinctive facet of healthcare delivery [2].

Non-registered practitioners encompass various definitions; however, the most widely accepted one pertains to individuals who are not registered or regulated by the local health authority. In Hong Kong, the majority of healthcare professionals, including medical doctors, Chinese medicine practitioners, nurses, chiropractors, physiotherapists, and other allied health professionals, are required to be registered with the Department of Health under corresponding legislation. Additionally, healthcare professionals who are not subject to statutory registration in Hong Kong, such as speech therapists, are regulated by accredited society-based registration [3,4]. Without these regulations, practitioners are deemed to be non-registered practitioners, such as massage therapists.

Spinal manipulative therapy (SMT) is a technique that involves applying a thrust or impulse to the spinal vertebrae. This treatment technique is commonly employed by various disciplines, including physical therapists, osteopaths, and chiropractors, to address spinal conditions [5,6]. SMT is recognized for its safety and effectiveness in managing both neck and lower back conditions [7-10], and it is recommended by multiple clinical practice guidelines [10-12].

SMT is deemed safe only when administered by a registered practitioner following a thorough examination, which may include orthopedic, radiological, and even medical laboratory investigations. This comprehensive approach aims to investigate and identify the underlying cause while ruling out serious pathological conditions [13].

Without a comprehensive examination, SMT carries the potential risk of serious adverse events, including fractures or cerebrovascular accidents [14-22]. Non-registered practitioners may lack the necessary knowledge to identify clinical red flags and the ability to request additional investigations or refer for emergency care. The absence of a thorough examination could significantly elevate the risk of harm to patients who opt for SMT [23].

This study aimed to explore the types of injuries and adverse events resulting from SMT administered by non-registered practitioners, from the perspective of a registered chiropractor. Additionally, it seeks to describe the subsequent actions taken to manage the patient. By gaining insights into these aspects, we can further understand the characteristics of non-registered practitioners and enhance the delivery of chiropractic care.

## Materials and methods

Design

The study utilized cross-sectional survey study to explore the chiropractors’ perceptive towards non-registered practitioner using spinal manipulation to patient.

Ethical considerations

The study received approval from the ethics committee of the Chiropractic Doctors' Association of Hong Kong (Causeway Bay, Hong Kong; IRB ID: CDA20231210), and written consent was waived since consent was implied by the return of the completed survey. To ensure respondent anonymity, names and other identifying information were not collected.

Setting 

The target population of this study comprises individuals currently registered and practicing chiropractic in Hong Kong.

Instrument

A self-developed comprehensive survey questionnaire with reference to a previous study was formulated in English to investigate chiropractors' perceptions towards non-registered practitioners utilizing SMT [13]. The questionnaire consisted of 17 questions for exploring chiropractors' practices and views on non-registered practitioners utilizing SMT.

Data sources

Data for this investigation were acquired through the distribution of a survey instrument implemented on the Google Forms platform. The survey was distributed to all registered chiropractors within Hong Kong, who were reached via their affiliated chiropractic associations. Official invitations to participate in the study were disseminated electronically, utilizing established communication channels maintained by the respective chiropractic associations. The completed survey instruments served as the exclusive source of data for this study. Subsequent to data collection, the data were extracted and compiled into a spreadsheet format to facilitate further analysis.

Bias 

Prior to full distribution, the survey underwent a pilot test with 10 chiropractors to verify question clarity and estimate completion time. All participants finished the survey within 10 minutes, and the pilot confirmed the questions' clarity, requiring no modifications.

Sample size

To ensure sufficient data collection, a rule-of-thumb estimation was applied, estimating five respondents needed per survey question. Based on this approach, a sample size of 100 participants was targeted for the study. Given that there were 346 registered chiropractors under the Hong Kong Chiropractors Council [24], and previous local survey studies collected 80 and 151 responses [13,25], this sample size was considered achievable.

Analysis

The data extracted from the questionnaires was analyzed using Microsoft Excel (Microsoft® Corp., Redmond, WA). Frequency tables and descriptive statistics were employed to examine the current chiropractors' perceptions of non-registered practitioners utilizing SMT.

## Results

Result 

The survey was administered online through a Google Forms link from January 26, 2024, to March 20, 2024. Out of the 161 responses received, 27 were incomplete, resulting in 134 valid responses (83.2% completion rate).

Respondent information

The survey revealed a diverse range of experience among chiropractors. A total of 12.7% (n=17) had less than a year of experience, while 27.6% (n=37) had been practicing for one to five years. Another 25.4% (n=34) had 5-10 years of experience, and the most experienced group (34.3%, n=46) had been practicing for over 10 years. In terms of practice type, the majority (57.5%, n=77) worked in solo settings, while 42.5% (n=57) operated in group environments.

The survey also investigated how often chiropractors encountered patients treated by non-registered practitioners in the past year. A significant portion (78.3%, n=105) reported encountering such patients on multiple occasions, ranging from six to more than 20 times in the past year. A breakdown of these encounters revealed that 2.2% (n=3) reported never encountering such patients, while 19.4% (n=26) saw these patients one to five times. Additionally, 17.9% (n=24) and 29.1% (n=39) reported encountering them 6-10 and 11-20 times, respectively. The most frequent encounters were reported by 31.3% (n=42) of chiropractors, who indicated seeing such patients more than 20 times in the past year. Table 1 presents the respondents’ practicing information.

**Table 1:** Respondents practicing information

Respondents		n=134 (%)
Years of experience		
	Less than 1 year	17 (12.7)
	1–5 years	37 (27.6)
	5–10 years	34 (25.4)
	More than 10 years	46 (34.3)
Type of practice		
	Group practice	57 (42.5)
	Solo Practice	77 (57.5)
Encounter patient who previous treated by non-registered practitioners in the past year		
	None	3 (2.2)
	1-5	26 (19.4)
	6-10	24 (17.9)
	11-20	39 (29.1)
	More than 20	42 (31.3)

Experiences with non-registered practitioners utilizing SMT

The most common complaints reported by patients seeking treatment from unlicensed practitioners were back pain, neck pain, headaches or migraines, and joint pain. Among these, back pain was the most frequently reported complaint, mentioned by 97% (n=130) of the respondents. This was followed by neck pain at 85.1% (n=114), joint pain at 73.9% (n=99), and headaches or migraines at 65.7% (n=88).

Unlicensed practitioners employed a variety of treatments for these patients. The most common treatment reported was massage therapy, used by 92.5% (n=124) of the respondents. Machine-assisted treatment was the next most frequently reported, used by 76.1% (n=102), followed by exercise or stretching therapy at 73.4% (n=97). Dietary supplements were reported as a treatment option by 45.5% (n=61), while acupuncture was the least frequently used treatment, mentioned by 15.7% (n=21). Table 2 illustrates the most common complaints and treatments reported by patients seeking care from unlicensed practitioners.

**Table 2:** Most common complaints and treatments reported by patients seeking care from unlicensed practitioners

Respondents		n=134 (%)
Most common complaint reported by patients seeking treatment from unlicensed practitioners		
	Back Pain	130 (97)
	Neck Pain	114 (85.1)
	Headaches/Migraines	88 (65.7)
	Joint Pain	99 (73.9)
Other treatments used by unlicensed practitioners on patients		
	Massage therapy	124 (92.5)
	Exercise/Stretching therapy	97 (73.4)
	Dietary supplements	61 (45.5)
	Machine-assisted treatment	102 (76.1)
	Acupuncture	21 (15.7)

Of the respondents, 91% (n=122) observed adverse effects or complications in patients who had received treatment from unlicensed practitioners before coming to their clinic. Meanwhile, 6% (n=8) were not sure, and 3% (n=4) did not observe any adverse effects.

Among those who observed adverse effects, the most frequently reported type was an aggravation of existing symptoms (95.5%, n=128). Other common adverse effects included new or unexpected complaints (82.8%, n=111) and delayed recovery (73.9%, n=99). Regarding progress under the respondents' care, 91% (n=122) of patients who had previously received treatment from unlicensed practitioners showed improvement in their condition, 6% (n=8) were unsure of the progress, and 3% (n=4) reported no progress.

For those patients who experienced adverse events, various additional interventions were required. The most common intervention was imaging (97%, n=130), followed by medication (58.2%, n=78), specialist referrals (59%, n=79), and surgery (29.1%, n=39). Only 1.5% (n=2) reported that no additional intervention was needed. Table 3 describes the additional interventions required for patients experiencing adverse events after treatment by unlicensed practitioners.

**Table 3:** Additional interventions required for patients experiencing adverse events after treatment by unlicensed practitioners

Intervention	n=134 (%)
Imaging	130 (97)
Medication	78 (58.2)
Specialist Referrals	79 (59.0)
Surgery	39 (29.1)
No Additional Intervention	2 (1.5)

The average number of treatment sessions required for complete recovery from adverse events varied. A total of 27% (n=36) of patients required 20-30 sessions, 25.4% (n=34) required 10-20 sessions, and 20.9% (n=28) needed more than 30 sessions. Meanwhile, 24.1% (n=32) achieved recovery within 3-10 sessions, and a small percentage, 3% (n=4), recovered within one to two sessions.

Perception of treatment by non-registered practitioners

In response to whether any of the adverse events observed could have been potentially avoidable if treated by a properly trained provider, 99.2% (n=133) of respondents agreed, while only 0.8% (n=1) believed they were not avoidable.

Regarding the types of adverse events deemed most preventable with appropriate training and certification, the most frequently identified issues included soft tissue strains/sprains and disc injuries, each noted by 85.1% (n=114) of respondents. This was closely followed by an inability to properly screen for red/yellow flags (90.3%, n=121), the use of inappropriate manipulation forces on the patient (88.8%, n=119), and the exacerbation of pre-existing conditions (83.6%, n=112).

Other commonly mentioned preventable events included neurological impairments (82.8%, n=111), poor communication of risks, benefits, and alternatives (69.7%, n=94), and vertebral fractures (66.4%, n=89). Failure to refer complex cases beyond the provider's scope was considered the least preventable, noted by 38.8% (n=52) of respondents. Table 4 shows adverse events considered most preventable with appropriate training and certification according to respondents.

**Table 4:** Adverse events considered most preventable with appropriate training and certification by respondents

Adverse Event	n=134 (%)
Inability to Properly Screen for Red/Yellow Flags	121 (90.3)
Use of Inappropriate Manipulation Forces	119 (88.8)
Soft Tissue Strains/Sprains	114 (85.1)
Disc Injuries	114 (85.1)
Exacerbation of Pre-existing Conditions	112 (83.6)
Neurological Impairments	111 (82.8)
Poor Communication of Risks, Benefits, and Alternatives	94 (69.7)
Vertebral Fractures	89 (66.4)
Failure to Refer Complex Cases Beyond the Provider's Scope	52 (38.8)

Respondents identified several key risks associated with spinal manipulation performed by unlicensed practitioners. The most frequently cited risk was a lack of appropriate training and certification (46.3%, n=62), followed by an inability to recognize red flags (25.4%, n=34). Poor technique or forceful manipulation was also noted as a significant risk (16.4%, n=22). Other concerns included treating complex issues beyond the practitioner's scope (9%, n=12) and inadequate screening practices (3%, n=4).

The survey also identified patient populations considered most at risk when seeking care from unlicensed practitioners. The most frequently cited at-risk populations included geriatric patients (89.6%, n=120), patients with complex medical histories or comorbidities (86.6%, n=116), and patients seeking care for traumatic injuries or acute pain (79.9%, n=107). Pediatric or adolescent patients (72.4%, n=97) and pregnant women (71.6%, n=96) were also identified as highly vulnerable groups.

Other at-risk populations noted by respondents included patients with compromised immune systems or bone health (67.2%, n=90), patients with poor health literacy (63.4%, n=85), and those seeking alternative options for chronic or terminal conditions (51.5%, n=69). Smaller percentages of respondents identified low-income patients (18.7%, n=25), those without health insurance (16.4%, n=22), and individuals lacking access to primary care (17.2%, n=23) as particularly vulnerable. Table 5 describes the patient populations considered most at risk when seeking care from unlicensed practitioners, as reported by respondents.

**Table 5:** Patient populations most at-risk when seeking care from unlicensed practitioners

At-Risk Population	n=134 (%)
Geriatric Patients	120 (89.6)
Patients with Complex Medical Histories or Comorbidities	116 (86.6)
Patients Seeking Care for Traumatic Injuries or Acute Pain	107 (79.9)
Pediatric or Adolescent Patients	97 (72.4)
Pregnant Women	96 (71.6)
Patients with Compromised Immune Systems or Bone Health	90 (67.2)
Patients with Poor Health Literacy	85 (63.4)
Patients Seeking Alternative Options for Chronic/Terminal Conditions	69 (51.5)
Low-Income Patients	25 (18.7)
Individuals Lacking Access to Primary Care	23 (17.2)
Those Without Health Insurance	22 (16.4)

Respondents identified several common reasons patients provided for seeking treatment from unlicensed practitioners. The most frequently cited reasons were a lack of awareness about licensed care (90.3%, n=121), testimonial social media advertisements (82.1%, n=110), and cost considerations (80.6%, n=108). Other reasons included direct-to-consumer marketing and advertising (75.4%, n=101) and a lack of awareness that licensed providers are required to have proper credentials (70.1%, n=94).

Additional reasons provided by respondents included recommendations from friends or family (35.8%, n=48), dissatisfaction with previous healthcare providers (25.4%, n=34), and a lack of referrals from primary care providers (25.4%, n=34). Smaller percentages cited co-location with other complementary therapies (15.7%, n=21) as a factor. Table 6 illustrates common reasons patients seek treatment from unlicensed practitioners.

**Table 6:** Common reasons patients seek treatment from unlicensed practitioners

Reason for Seeking Treatment	n=134 (%)
Lack of Awareness About Licensed Care	121 (90.3)
Testimonial Social Media Advertisements	110 (82.1)
Cost Considerations	108 (80.6)
Direct-to-Consumer Marketing and Advertising	101 (75.4)
Lack of Awareness That Licensed Providers Are Required to Have Credentials	94 (70.1)
Recommendations from Friends or Family	48 (35.8)
Dissatisfaction with Previous Healthcare Providers	34 (25.4)
Lack of Referrals from Primary Care Providers	34 (25.4)
Co-location with Other Complementary Therapies	21 (15.7)

The survey also explored the most common reasons patients who had previously received treatment from unlicensed practitioners chose to seek care from licensed providers. The most frequently reported reason was the worsening of symptoms (66.4%, n=89). Other reasons included a lack of improvement in their condition (21.6%, n=29) and dissatisfaction with the results from unlicensed practitioners (9.7%, n=13). A small number of patients sought care for a second opinion (0.7%, n=1) or following a referral from another healthcare professional (1.5%, n=2). Table 7 shows the reasons patients seek care from licensed providers after treatment by unlicensed practitioners.

**Table 7:** Reasons patients seek care from licensed providers after treatment by unlicensed practitioners

Reason for Seeking Care from Licensed Providers	n=134 (%)
Worsening of Symptoms	89 (66.4)
Lack of Improvement in Their Condition	29 (21.6)
Dissatisfaction with Results from Unlicensed Practitioners	13 (9.7)
Seeking a Second Opinion	1 (0.7)
Referral from Another Healthcare Professional	2 (1.5)

All respondents (100%, n=134) agreed that there is a need for increased public awareness about the importance of seeking treatment from licensed healthcare professionals for pain conditions. No respondents expressed neutrality, disagreement, or uncertainty on this topic.

## Discussion

The findings of this cross-sectional survey provide important insights into the perceptions of registered chiropractors in Hong Kong regarding the use of SMT by non-registered practitioners. The survey results highlight several critical concerns, including the frequency of patient encounters with adverse outcomes, the types of injuries observed, and the perceived risks associated with SMT performed by unlicensed providers. These findings underscore significant challenges related to patient safety and emphasize the need for increased public awareness and stronger regulatory measures.

A substantial proportion of respondents (98.8%) reported encountering patients who had previously received treatment from non-registered practitioners and subsequently experienced adverse effects, such as aggravation of existing symptoms, new complaints, and delayed recovery. More severe adverse events, including soft tissue strains, disc injuries, and vertebral fractures, were also observed by the respondents, consistent with findings from previous studies [14-22]. The various adverse events indicate a potential risk associated with receiving SMT from non-registered practitioners who lack appropriate training and certification. Such practitioners may be unable to recognize clinical red flags or apply proper manipulation techniques, increasing the risk of harm to patients [23].

The data suggest that many of these adverse events might have been preventable if patients had been treated by properly trained and certified providers. SMT is generally considered safe when performed following thorough screening and examination to rule out underlying pathology [13]. However, specific populations - such as geriatric patients, those with complex medical histories or comorbidities, and those seeking care for traumatic injuries or acute pain - are particularly vulnerable due to their complex health needs. These groups may have contraindications to certain types of manual therapy, making them especially susceptible to adverse outcomes [26]. Non-registered practitioners may lack the requisite skills to adequately assess and manage these high-risk populations, increasing the likelihood of adverse events [23]. This is further supported by the survey results, which show a tendency among respondents to use imaging as an additional intervention, emphasizing the importance of thorough examination before utilizing SMT to minimize risks.

Moreover, the need for additional interventions such as imaging, medication, and specialist referrals demonstrates the significant burden on healthcare resources caused by complications arising from treatments administered by unlicensed practitioners. The wide variation in the number of treatment sessions required for full recovery further indicates that adverse outcomes can lead to prolonged recovery periods, increased patient costs, and added strain on the healthcare system [13,27].

This study also provides insights into why patients may choose to seek treatment from non-registered practitioners. The most commonly cited reasons included a lack of awareness about licensed care, cost considerations, and the influence of social media advertisements. These findings point to a gap in public knowledge regarding the qualifications and standards required for safe and effective SMT, as well as the impact of marketing strategies employed by non-registered practitioners [28,29].

Patients who experienced worsening symptoms, lack of improvement, or dissatisfaction with their care from non-registered practitioners often sought care from licensed providers. This underscores the importance of patient education on the risks associated with unlicensed practitioners and the benefits of seeking care from properly trained professionals.

These findings have several implications for both clinical practice and policy. First, there is a clear need for enhanced public education and awareness campaigns to inform patients about the importance of seeking care from registered practitioners, particularly for conditions that require specialized expertise such as SMT. This may involve efforts by professional associations, public health authorities, and other stakeholders to provide accurate information about the risks associated with unlicensed practice and the benefits of credentialed care.

Second, the study underscores the importance of enhanced regulatory oversight while recognizing that regulation alone may not be the only solution. While stricter policies and clearer standards for SMT use are necessary, there is also an opportunity for professional associations to play a proactive role in public education. These bodies can help inform the public about the advantages of consulting licensed professionals compared to non-registered practitioners.

While this study provides valuable insights, it also has limitations. First, the cross-sectional design makes it impossible to establish causal relationships between non-registered practice and adverse events, as no detailed investigations or interviews with non-registered practitioners were conducted. The data rely on self-reported information from registered chiropractors, which introduces not only recall bias but also the potential for social desirability bias, where adverse events could be over-reported to discredit non-registered competitors in the same market. Additionally, the study did not collect its own data, raising the possibility of information bias. The sample was one of convenience, and while adequate for a preliminary investigation, it may not capture the full range of experiences among chiropractors in Hong Kong. Future research should consider a broader and more diverse sample and longitudinal designs to better assess the long-term outcomes of non-registered SMT practices.

## Conclusions

This study reveals significant concerns among registered chiropractors regarding the safety and efficacy of SMT performed by non-registered practitioners in Hong Kong. Reports from chiropractors suggest potential adverse events, particularly among vulnerable patient groups, which indicate the need for further investigation. To ensure patient safety, enhanced public education and regulatory measures could be explored. Addressing these concerns through targeted interventions, public awareness campaigns, and stricter enforcement of professional standards may help mitigate the risks associated with unlicensed practice and improve overall healthcare outcomes in Hong Kong.
